# Development and pyrosequencing analysis of an in-vitro oral biofilm model

**DOI:** 10.1186/s12866-015-0364-1

**Published:** 2015-02-10

**Authors:** James O Kistler, Manuel Pesaro, William G Wade

**Affiliations:** Centre for Immunology and Infectious Disease, Barts and The London School of Medicine and Dentistry, Queen Mary University of London, London, UK; Symrise AG, Holzminden, Germany

**Keywords:** 16S rRNA, Bacteria, Saliva, Plaque, Microbiome, Microbiota

## Abstract

**Background:**

Dental caries and periodontal disease are the commonest bacterial diseases of man and can result in tooth loss. The principal method of prevention is the mechanical removal of dental plaque augmented by active agents incorporated into toothpastes and mouthrinses. In-vitro assays that include complex oral bacterial biofilms are required to accurately predict the efficacy of novel active agents *in vivo*. The aim of this study was to develop an oral biofilm model using the Calgary biofilm device (CBD) seeded with a natural saliva inoculum and analysed by next generation sequencing. The specific objectives were to determine the reproducibility and stability of the model by comparing the composition of the biofilms over time derived from (i) the same volunteers at different time points, and (ii) different panels of volunteers.

**Results:**

Pyrosequencing yielded 280,093 sequences with a mean length of 432 bases after filtering. A mean of 320 and 250 OTUs were detected in pooled saliva and biofilm samples, respectively. Principal coordinates analysis (PCoA) plots based on community membership and structure showed that replicate biofilm samples were highly similar and clustered together. In addition, there were no significant differences between biofilms derived from the same panel at different times using analysis of molecular variance (AMOVA). There were significant differences between biofilms from different panels (AMOVA, *P* < 0.002). PCoA revealed that there was a shift in biofilm composition between seven and 14 days (AMOVA*, P* < 0.001). *Veillonella parvula*, *Veillonella atypica*/*dispar*/*parvula* and *Peptostreptococcus stomatis* were the predominant OTUs detected in seven-day biofilms, whilst *Prevotella oralis*, *V. parvula* and *Streptococcus constellatus* were predominant in 14-day biofilms.

**Conclusions:**

Diverse oral biofilms were successfully grown and maintained using the CBD. Biofilms derived from the same panel of volunteers were highly reproducible. This model could be used to screen both antimicrobial-containing oral care products and also novel approaches aiming to modify plaque composition, such as pre- or probiotics.

**Electronic supplementary material:**

The online version of this article (doi:10.1186/s12866-015-0364-1) contains supplementary material, which is available to authorized users.

## Background

Dental caries and the periodontal diseases are the commonest bacterial diseases of man and can result in the loss of the teeth and their associated function, and are also significant risk factors for disease at other body sites, particularly cardiovascular disease [1]. Treatment of oral diseases is expensive and efforts have therefore focused on prevention, particularly through the use of mouthrinses and toothpastes containing active agents that control the bacteria found in dental plaque. The evaluation of novel active agents and formulations in humans is time-consuming and difficult and toxicological data may not be available for new compounds. Consequently, attempts have been made to develop in-vitro assays that accurately predict efficacy *in vivo* [2,3]. These assays enable researchers to perform preliminary screening of active agents in order to identify candidates for subsequent testing in clinical trials, wherein the therapeutic effects, as well as issues such as substantivity in the oral cavity [4], can be determined.

Many existing in-vitro assays cultivate oral bacteria for testing in planktonic suspension but it has been shown that bacteria naturally form biofilms [5]. Bacteria in biofilms exhibit greater resistance to antimicrobial agents than cells growing in planktonic culture [6,7]. In addition, the composition of oral biofilms is typically highly complex and includes a substantial number of species which have yet to be cultivated [8]. Recent deep sequencing studies have, for example, detected hundreds of species in dental plaque samples from individual subjects [9-11]. Although some in-vitro oral biofilm assays have been developed, these have typically used relatively simple defined inocula [12-15]. Given the high richness and diversity of oral biofilms, it would be preferable to use natural inocula in order to more accurately represent the in-vivo ecosystem.

One in-vitro system that has been previously developed and used to grow bacteria as biofilms is the Calgary biofilm device (CBD) [16]. In this system, biofilms are grown on pegs protruding from the lid of a 96-well plate. The pegs are immersed in a growth medium that can easily be replaced by transferring the lid to a new baseplate, thereby enabling the long-term growth of biofilms. The CBD was originally developed to determine the susceptibility of bacterial biofilms to antibiotics [16] for applications such as medical device-related infections, and is therefore commercially available as the ‘Minimum Biofilm Eradication Concentration’ (MBEC) assay (Innovotech, Canada). Previous work has demonstrated that uniform biofilms with reproducible total viable counts can be obtained when using simple defined bacterial inocula [17]. The CBD has also been used to examine the interactions among five common oral species when grown anaerobically for up to 36 hours [18]. Using quantitative-PCR, the authors showed that *Porphyromonas gingivalis* cell counts increased when grown together with a *Veillonella* sp., *Fusobacterium nucleatum*, or *Aggregatibacter actinomycetemcomitans*, suggesting mutualism between these species.

The overall aim of this study was to develop an oral microbial biofilm model derived from a natural inoculum: the saliva of healthy individuals, using the CBD. The biofilms thus generated will be suitable for assessing the impact of different oral care product components on oral biofilm composition. The specific aims of this study were to determine the reproducibility, stability and variability of the model by using pyrosequencing of partial 16S rRNA genes to compare the composition of the biofilms over time derived from saliva from (i) the same individuals at different time points and (ii) from different panels of volunteers.

## Methods

### Participants

Ethical approval for this study was granted by Queen Mary University of London Ethics of Research Committee (reference no. QMREC2013/58). Informed consent was obtained from all of the individuals who participated. All of the participants were between 18 and 65 years of age and were medically healthy volunteers who were staff or postgraduate students at Queen Mary University of London. Any subjects with systemic conditions that may have affected their immune or inflammatory status were excluded from the study. A total of 18 subjects participated in the study.

### Sample collection

Un-stimulated saliva samples were obtained from the participants by expectoration into sterile universal tubes. Saliva was collected between approximately 14:30 and 15:00 on the days that the biofilms were to be inoculated. Participants were grouped into panels of six and their saliva samples pooled together in equal volumes: 1 ml was used from each individual to produce a 6 ml pooled sample. Pooled saliva was placed on ice and processed within an hour. One panel was sampled at three different time points, a week apart, and two panels were sampled at one time point.

### Inoculation of the Calgary biofilm device

The saliva was vortexed for 15 s and 200 μl was pipetted per well of a 96-well microplate, up to the required number of wells. Wells around the outside of the microplate were not used. The lid of the CBD was fitted onto the microplate so that the hydroxyapatite-coated pegs were bathed in the saliva. The CBD plate was then incubated at 37°C under anaerobic conditions (80% N_2_, 10% H_2_, and 10% CO_2_) for 18 hours, after which the lid was transferred to a new baseplate containing 200 μl of pre-reduced Brain Heart Infusion broth (Fluka Analytical) growth medium supplemented with hog gastric mucin (1 g/L), haemin (10 mg/L), and vitamin K (0.5 mg/L). The growth medium was changed after every 3.5 days of anaerobic incubation. Biofilms were harvested from half of the pegs after seven days and from the remaining half after 14 days.

### Removal of pegs and propidium monoazide treatment of samples

Pegs with biofilms were snapped off the lid with sterile pliers and washed by dipping into sterile phosphate-buffered saline (PBS) three times. All of the visible biofilm material was then removed using a sterile curette and suspended into 500 μl of sterile PBS. The material from three pegs was pooled to produce one sample for analysis, and three samples were processed for each incubation time. Each sample, and half of the original saliva sample, was subjected to propidium monoazide (PMA) treatment to prevent subsequent PCR amplification of extracellular DNA and DNA from dead or damaged cells [19]: 1.25 μl of PMA was added (at a final concentration of 50 μM) to the cells suspended in PBS and incubated in the dark with occasional shaking for 5 mins at room temperature. The samples were then exposed to light from a 500 W halogen lamp for 5 mins at a distance of 20 cm in order to form a covalent linkage between the PMA and the DNA. During the exposure time the samples were placed on ice to avoid excessive heating and subjected to occasional shaking. The samples were used for DNA extractions immediately after the PMA treatment.

### DNA extraction

DNA was extracted from the saliva and biofilm samples using the GenElute Bacterial DNA extraction kit (Sigma-Aldrich). Extractions were performed on both PMA-treated and -untreated aliquots (500 μl) of pooled saliva samples. DNA extraction was performed following the manufacturer’s instructions with an additional cell lysis step to increase the recovery of DNA from Gram-positive cells, in which samples were incubated in a 45 mg/ml lysozyme solution at 37°C for 30 mins.

### Molecular microbiological analysis

The bacterial composition of the biofilms and saliva was determined using 454 pyrosequencing of partial 16S rRNA genes as described previously [11], with some minor modifications. PCR amplification of a fragment of the 16S rRNA gene, approximately 500 bp in length covering the V1-V3 hypervariable regions, was performed for each DNA sample using composite fusion primers. The fusion primers comprised the broad-range 16S rRNA gene primers 27 FYM [20] and 519 R [21] along with Roche GS-FLX Titanium Series adapter sequences (A and B) for 454-pyrosequencing using the Lib-L emulsion-PCR method. The forward primers included previously described 12-base error-correcting Golay barcodes. PCR reactions were performed using Extensor Hi-fidelity PCR mastermix (Thermo-Scientific) along with the appropriate barcoded forward primer and the reverse primer. The PCR conditions were as follows: 5 mins initial denaturation at 95°C, followed by 25 cycles of 95°C for 45 s, 53°C for 45 s and 72°C for 45 s and a final extension of 72°C for 5 mins. PCR amplicons were then purified using the QIAquick PCR purification kit (Qiagen) according to the manufacturer’s instructions. The size and purity of the amplicons was checked using the Agilent DNA 1000 kit and the Agilent 2100 Bioanalyzer. Quantitation of the amplicons was performed by means of a fluorometric assay using the Quant-iT Picogreen fluorescent nucleic acid stain (Invitrogen). The amplicons were then pooled together at equimolar concentrations (1 × 10^9^ molecules/μl). Emulsion-PCR and unidirectional sequencing of the samples was performed using the Lib-L kit and the Roche 454 GS-FLX + Titanium series sequencer by the Department of Biochemistry, Cambridge University, Cambridge, UK.

The raw sequence data were deposited with the NCBI SRA database as accession SRP051689.

### Sequence analysis

Sequence analysis was performed using the ‘mothur’ software suite version 1.33 [22], following the 454 standard operating procedure [23] on mothur.org. The sequences were denoised using the AmpliconNoise algorithm [24], as implemented by mothur. Sequences that were less than 440 bases in length and/or had one of the following: >2 mismatches to the primer, >1 mismatch to the barcode regions, and homopolymers of >8 bases in length, were discarded. The remaining sequences were trimmed to remove primers and barcodes and aligned to the SILVA 16S rRNA reference alignment [25]. The UChime algorithm [26] was used to identify chimeric sequences, which were then removed from the dataset. Sequences were clustered into operational taxonomic units (OTUs) at a genetic distance of 0.015 using the average neighbour algorithm and identified using a Naïve Bayesian classifier [27] with the Human Oral Microbiome Database (HOMD) reference set (version 13). For those OTUs that could not be identified using the Bayesian classifier, representative sequences were obtained in mothur (the sequence with the smallest distance to all other sequences in that OTU) and identified using BLAST against the HOMD reference set (version 13). The possible alternatives for the species identification were then provided.

#### Analysis of alpha and beta diversity

The sequences for each sample were randomly sub-sampled to the same number (that of the sample with the lowest number of sequences: 3023) for the alpha and beta diversity analyses. The extent of sampling of the communities was assessed using Good’s non-parametric coverage estimator [28]. The diversity of the communities was calculated using Simpson’s inverse diversity index [29]. The beta-diversity of the samples was analysed using distance matrices generated using the Jaccard index and the thetaYC calculator [30]. The distance matrices were visualised using principal coordinates analysis (PCoA) plots generated in R (r-project.org).

#### Statistical analysis

Analysis of molecular variance (AMOVA) [31], as implemented in mothur, was used to determine if there were statistically significant differences in Jaccard index and thetaYC distances between saliva and biofilm samples and between biofilms from different time points, subject panels, and incubation times. The mean relative abundances of phyla, genera, and species-level OTUs in biofilms was determined as follows: The proportions of sequences assigned to a particular taxon were calculated for biofilms derived from different panels at a single time point only (one panel was sampled at three time points) after 7- and 14-day incubations. The PMA-treated saliva samples were used for statistical comparisons of the taxonomic composition of saliva to biofilms. To determine if there were significant differences in OTU richness and diversity between the incubation times, paired t-tests were performed in R.

## Results

### Pyrosequencing

A total of 232,757 sequences with a mean length of 432 bases were obtained for analysis after quality filtering, screening of the sequence alignment, and removal of chimeras. A mean of 5968 sequences (range: 3023–7637) were obtained per sample. One replicate biofilm sample derived from Panel 1 after seven days of incubation (P1_T3_7D_c) was not included in the sequencing run due to poor PCR amplification. The number of OTUs (clustered at a distance of 0.015) detected in individual biofilm and pooled saliva samples ranged between 195 and 391. The mean number of OTUs detected was 250 in the biofilms and 320 in the saliva samples. A table summarising the alpha diversity of the biofilms and saliva is shown in Additional file 1. The number of OTUs detected in the seven-day biofilms (mean = 270.4) was significantly higher (*P* < 0.003) than in the 14-day biofilms (mean = 230.4). However, there was no significant difference in diversity (Simpson’s inverse diversity index) between the seven- and 14-day biofilms.

### Reproducibility of biofilms and shifts in biofilm OTU composition over time

**Figure 1:**
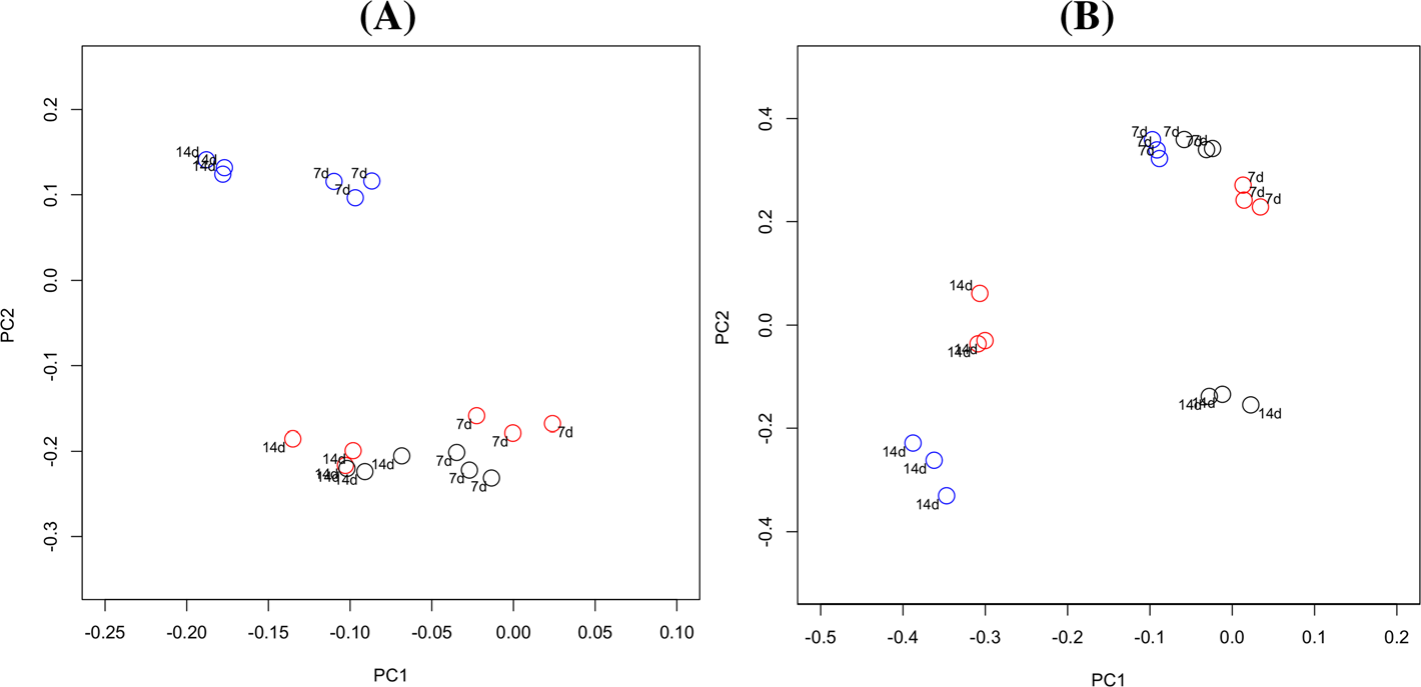
**Principal coordinates analysis of biofilms derived from different panels after different incubation times.** Plots are based on community membership using the Jaccard index **(A)** and community structure using the thetaYC calculator **(B)**. Blue - Panel 1; red - Panel 2; black - Panel 3. Labels indicate the incubation time. A: PC1 = 8.6% of variance, PC2 = 5.2% of variance. B: PC1 = 33.1% of variance, PC2 = 19.3% of variance.

**Figure 2:**
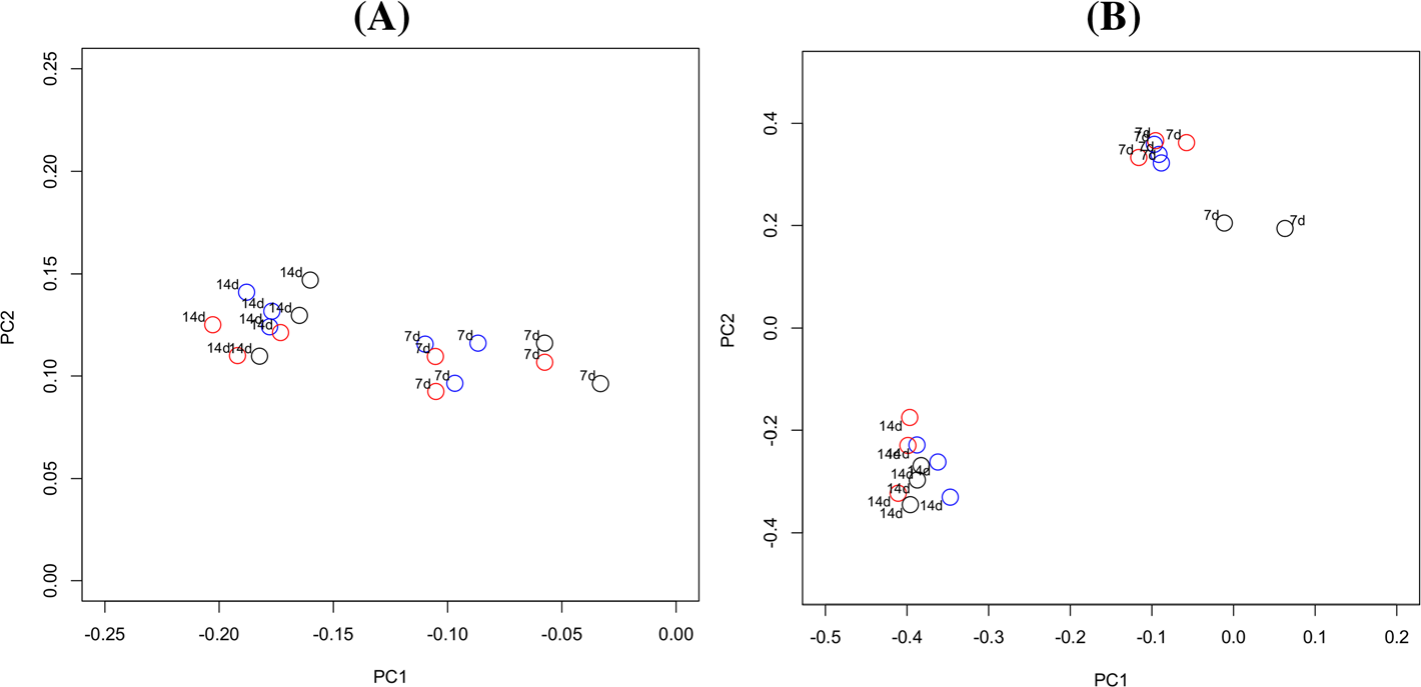
**Principal coordinates analysis of biofilm replicates from Panel 1 at different time points.** Plots are based on community membership using the Jaccard index **(A)** and community structure using the thetaYC calculator **(B)**. Blue - Time 1; red - Time 2; black - Time 3. A: PC1 = 8.6% of variance, PC2 = 5.2% of variance. B: PC1 = 33.1% of variance, PC2 = 19.3% of variance.

Comparison of the community membership and structure of biofilms using principal coordinates analysis (PCoA) plots indicated that replicate biofilm samples, derived from the same saliva pool after the same incubation time but harvested from different pegs, were highly similar and clustered together (Figure 1). In addition, biofilms derived from the same panel (Panel 1) at different times were similar (Figure 2) and AMOVA tests found there to be no statistically significant difference between the time points. However, there were significant differences in both the membership and structure of biofilms derived from the three different subject panels. The most significant differences by AMOVA were between biofilms from Panel 1 and Panel 3 (*P* = 0.001 for both membership and structure). Interestingly, PCoA indicated that the dissimilarity in community structure between panels was greater after 14 days than after seven days (Figure 1).

**Figure 3:**
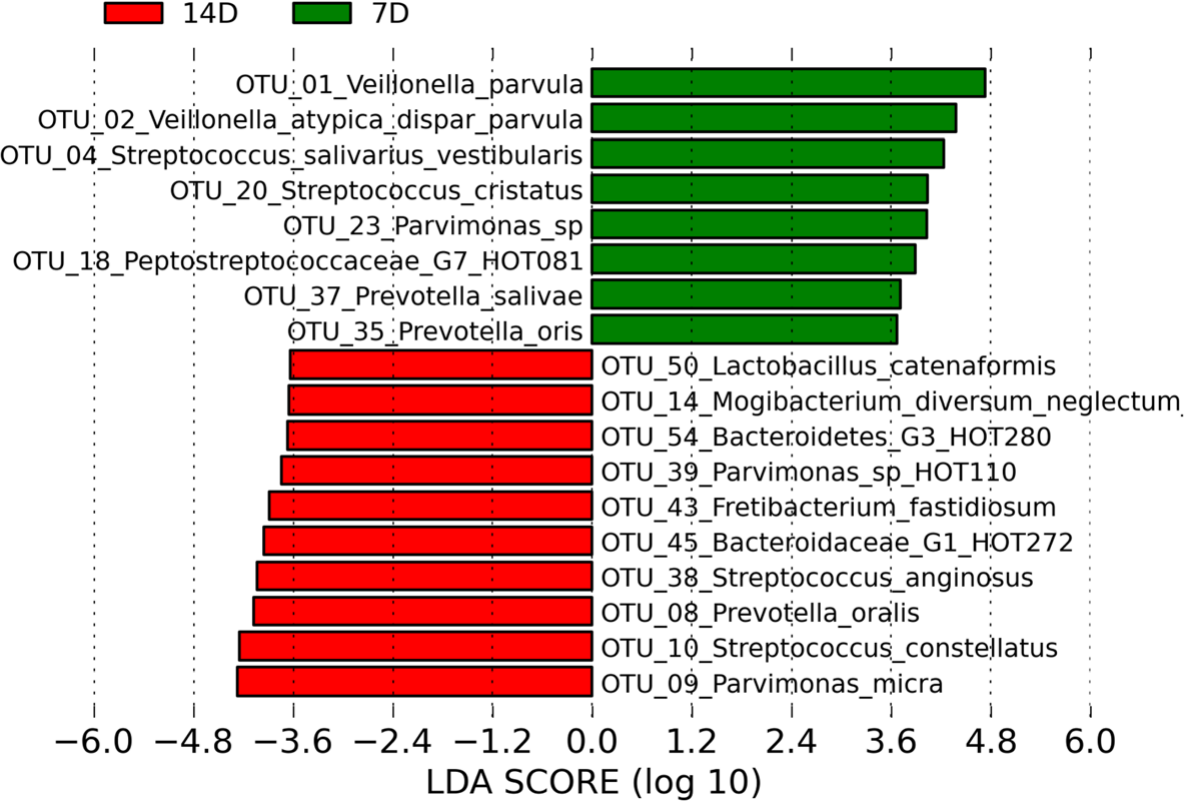
**Linear Discriminant Analysis Effect Size (LEfSe) analysis showing those OTUs that were significantly differentially abundant between seven- and 14-day incubation times, ranked by effect size (all LDA scores >3.5).**

There was a directional shift along the axes in the PCoA plots between seven and 14 days of incubation for biofilms derived from all three panels (Figures 1 and 2). AMOVA confirmed that there was a significant difference between the 7-day and 14-day incubations both in terms of membership and structure (*P* < 0.001 for both comparisons). Analysis using LEfSe identified a total of 74 OTUs that were significantly differentially abundant between incubation times. The identities of the OTUs with LDA effect size scores of >3.5 are shown in Figure 3. An OTU identified as *Veillonella parvula* was most strongly associated with the 7-day incubations, whilst *Parvimonas micra* was most strongly associated with the 14-day incubations.

Hierarchical cluster analysis showed that the biofilm samples clustered by panel in a dendrogram based on community membership (Additional file 2) and predominantly by incubation time in a dendrogram based on structure (Additional file 3).

### OTU-based comparisons of biofilms to saliva

**Figure 4:**
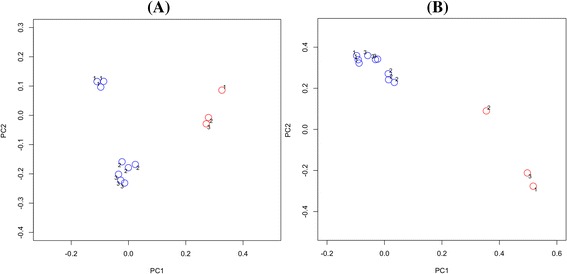
**Principal coordinates analysis of saliva and seven-day biofilms.** Plots are based on community membership using the Jaccard index **(A)** and community structure using the thetaYC calculator **(B)**. Blue - biofilms; red - saliva. Labels indicate the panel number. A: PC1 = 8.6% of variance, PC2 = 5.2% of variance. B: PC1 = 33.1% of variance, PC2 = 19.3% of variance.

**Figure 5:**
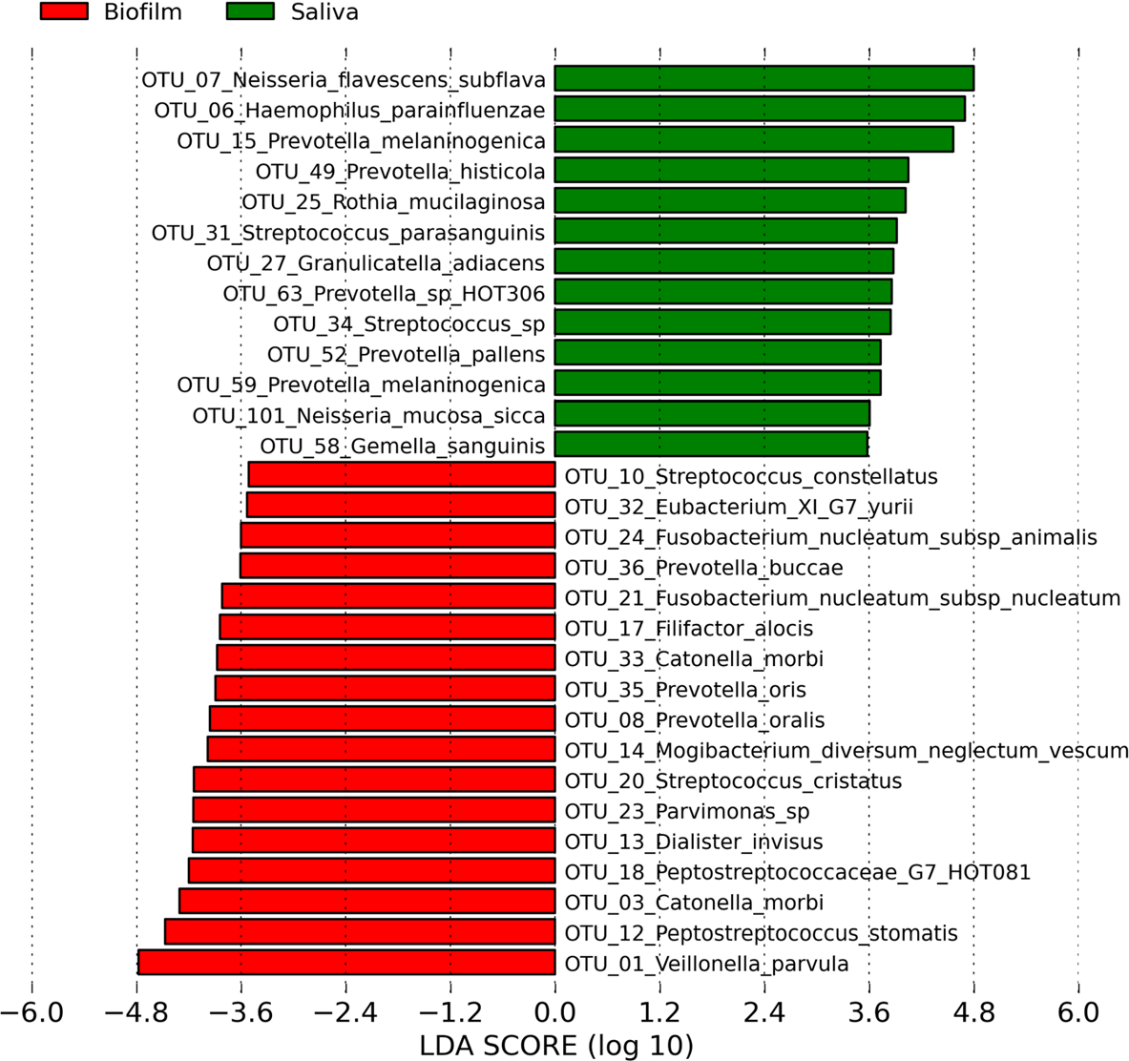
**Linear Discriminant Analysis Effect Size (LEfSe) analysis showing those OTUs that were significantly differentially abundant between saliva and seven-day biofilms, ranked by effect size (all LDA scores >3.5).**

Comparison of saliva samples with the biofilms using PCoA plots revealed a differing community membership and structure (Figure 4). AMOVA tests confirmed that there were significant differences in both the membership and structure of saliva compared to seven and 14-day biofilms (*P <* 0.001 for both comparisons). Analysis using LEfSe identified 112 OTUs that were significantly differentially abundant between saliva and biofilms that had been grown for seven days. A list of differentially abundant OTUs with LDA effect size scores of >3.5 is shown in Figure 5. An OTU identified as *Neisseria flavescens*/*subflava* was most strongly associated with saliva, whilst *Veillonella parvula* was most strongly associated with the biofilms.

### Comparison of PMA-treated and untreated saliva samples

Comparisons using PCoA showed that PMA-treated and untreated saliva were similar and samples clustered by the panel from which they were obtained, rather than by treatment (not shown). There were no significant differences in community membership or structure between PMA-treated and untreated saliva using AMOVA tests.

### Taxonomic composition of the biofilms

**Figure 6:**
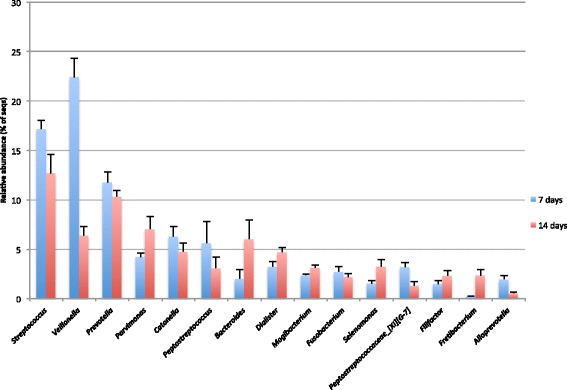
**Predominant genera detected in the biofilms.** The graph shows the mean relative abundances of genera that were detected in seven and 14-day biofilms derived from three panels. Genera shown are those with mean relative abundances of > 1%. Error bars show the standard error of the mean (SEM).

The predominant phyla detected in all of the biofilms in order of mean relative abundance were: *Firmicutes*, *Bacteroidetes*, *Synergistetes*, *Fusobacteria*, *Proteobacteria* and *Actinobacteria.* Other phyla that were detected in minor relative proportions, and not in every sample, included: SR1, *Spirochaetes*, TM7 and *Tenericutes*. A total of 102 genera were detected in the biofilms, the most abundant of which were: *Veillonella, Streptococcus* and *Prevotella* in seven-day biofilms, and *Streptococcus, Prevotella* and *Parvimonas* in 14-day biofilms. Figure 6 shows the relative abundances of the predominant genera detected in the biofilms after seven and 14 days incubation. The relative abundances of the predominant genera detected in the saliva samples, from which biofilms were derived, are shown in Additional file 4. A table detailing all of the taxa identified down to the species level, and their relative proportions in individual biofilm and saliva samples, can be found in Additional file 5.

## Discussion

This study has demonstrated that complex oral biofilms derived from a natural saliva inoculum can be successfully grown and maintained using the CBD. The results showed that the biofilms had a richness and diversity close to that of the pooled saliva inocula, with a mean of 250 species-level OTUs detected per biofilm sample compared to a mean of 320 in saliva. There was a significant difference in community membership and structure between the saliva and the biofilms, with some OTUs detected in saliva not present in the biofilms. This is not surprising because the bacterial composition of saliva is known to differ to that of dental plaque [32]. Saliva, however, does include representatives of the various surfaces found in the mouth and is thus a useful inoculum for biofilms. Hydroxyapatite-coated pegs were used for the purpose of mimicking the teeth in order to obtain biofilms with a similar composition to plaque. Another possible reason for differences between the inocula and the biofilms is that specific nutrients or growth factors required by certain species could have been absent in the growth medium used. A Brain-Heart Infusion (BHI) based medium was chosen in this study because it has been successfully used to cultivate a broad range of fastidious and non-fastidious oral bacteria [33]. The BHI was supplemented with mucin, vitamin K, and haemin, as some oral species grow poorly or not at all in the absence of one or more of these substances. For example, a number of black-pigmented species of *Prevotella* and *Porphyromonas* require haemin and vitamin K for growth [34]. In addition, hog gastric mucin, a high molecular weight glycoprotein, has been shown to support the growth of mixed communities of oral bacteria when used as the principal source of carbon and energy [35]. Future work could investigate the use of different media, such as an artificial saliva-based medium, with this model. Another reason that certain salivary species may have been lost is that the anaerobic atmosphere in which the CBD was incubated would have selected against the growth of aerobic species. The absence of host immune cells and molecules in the second phase of growth may also have had an impact on the community composition. Nevertheless, a highly diverse community of oral bacteria was maintained which included the genera known to be predominant in plaque and also a variety of fastidious and uncultivated taxa, such as un-named *Bacteroidetes*, *Lachnospiraceae*, *Clostridiales* and *Peptostreptococcaceae* species.

Both saliva and biofilm samples were treated with propidium monoazide prior to DNA extraction to avoid detection of bacterial cells that were non-viable; this method has been shown to prevent PCR amplification of DNA from dead or damaged cells [19]. Interestingly, there was no significant difference between PMA-treated and untreated saliva in terms of community membership and structure. This suggests that the vast majority of taxa detected in the saliva samples were viable. This could be due to the rapid processing of the samples performed in order to avoid loss of cell viability. In addition, human saliva has been shown to contain DNAse I produced by the parotid glands [36], which could rapidly break down extracellular bacterial DNA from dead cells.

The ability to grow biofilms with a richness and diversity close to that detected in oral habitats, and that include previously uncultivated taxa, is a major strength of this model over others. For instance, the well-established Zürich biofilm model [14] has been developed with defined inocula consisting of five, or more recently, 10 cultivable species [37]. Defined biofilms consisting of a low number of selected cultivable species are less representative of the in-vivo ecosystem and may, therefore, be less accurate in predicting the efficacy of an active agent. Another study recently reported the development of an in-vitro biofilm model in which a natural saliva inoculum was used and the composition of the samples determined by pyrosequencing of 16S rRNA genes [38]. Whilst the authors also reported a high microbial diversity, the incubation times used were relatively short (up to 48 hours) and this may explain why certain slow-growing oral taxa, including *Fretibacterium* spp. and *Tannerella* spp. were not detected, whilst streptococci were dominant with *S. vestibularis* constituting approximately 40% of the communities.

The taxonomic composition of the biofilms grown in this study was similar to that of dental plaque. *Streptococcus*, *Veillonella* and *Prevotella* were the predominant genera detected in the biofilms, all of which have been shown to be major constituents of plaque [39,40]. The OTU detected with the highest mean relative abundance in the biofilms was *Veillonella parvula,* which was also detected with the highest rank abundance in an extensive cloning and Sanger sequencing study of the human oral microbiome [8]. In addition, periodontitis-associated species, including the ‘red complex’: *Porphyromonas gingivalis, Treponema denticola, Tannerella forsythia,* and the Gram-positive anaerobes *Filifactor alocis* and *Parvimonas micra,* were all detected in the biofilms. These organisms have been strongly associated with deep periodontal pockets in individuals with severe chronic periodontitis [9,41]. In the CBD model, *P. micra* was the organism most strongly associated with 14-day biofilms. Species that had a significantly lower relative abundance in 14-day biofilms than 7-day biofilms included the streptococcal species *S. cristatus* and *S. salivarius* / *vestibularis,* which have previously been associated with health [9,42]*.* Species among the genera *Neisseria* and *Rothia* were detected at only very low proportions in the biofilms, despite being abundant in saliva. This is likely explained by the anaerobic incubation of the CBD, as these organisms grow optimally under aerobic conditions [43,44]. It has been shown that the redox potential (Eh) of dental plaque rapidly falls as the biofilm develops *in vivo* [45]. In addition, experimental gingivitis studies have shown that plaque accumulating in the absence of oral hygiene supports the growth of increasing numbers of anaerobic species, many of which are gingivitis-associated [11,33]. This study aimed to grow biofilms that were similar in composition to biofilms that would develop naturally *in vivo* without oral hygiene intervention, and anaerobic incubation was chosen in order to reproducibly obtain a biofilm typical of mature plaque. However, future work could examine the composition of CBD oral biofilms grown under aerobic conditions and compare them to those grown anaerobically. If using the biofilm model to screen antimicrobial agents or oral care product components, it would be useful to grow the biofilms under both aerobic and anaerobic conditions in order to determine the effect(s) of a given substance on as diverse a range of oral taxa as possible. Future studies could also compare the similarity of the in-vitro biofilms to dental plaque biofilms that form naturally *in vivo* in the same individuals abstaining from oral hygiene. This would further confirm that this model generates oral biofilms that are representative of those formed *in vivo*.

The results of this study showed that the bacterial composition of the biofilms was highly reproducible for sample replicates from different pegs derived from the same saliva pool and incubated for the same length of time. Moreover, the biofilms were similar in composition when derived from the same panel at different time points. After 14 days of incubation, when dissimilarity in the biofilms might have been expected to increase, the biofilms from the same panel clustered closely in the PCoA plots. The differences between biofilms derived from different panels was not surprising given the high inter-individual variation in bacterial diversity found in the normal human oral microbiome [46,47], although, an attempt was made to reduce this variability by pooling saliva from six individuals for use as the inoculum. Hierarchical clustering of the biofilm samples in dendrograms indicated that the panel was the primary determinant of community membership, but that incubation time had a stronger influence on community structure. This is likely to be because the relative abundances of OTUs would be expected to change over time as the biofilms mature. Due to the differences in both membership and structure of biofilms derived from different panels, the same panel should be used to provide the saliva inoculum in future studies that aim to compare biofilms grown under different conditions, or after exposure to different challenges e.g. antimicrobial agents. The reproducible biofilms that can be obtained using this model will enable relatively small changes in bacterial composition to be detected. This will be particularly useful for assessing the impact of oral care products that aim to manipulate or alter, rather than eradicate, plaque. This includes active agents or bacteriocin-producing probiotics that target particular taxa, or prebiotics that could promote the growth of health-associated bacteria. In the case of probiotics, the model could also be useful in helping to predict whether or not a particular strain is likely to colonise and persist within oral biofilms. In addition to determining changes in the community composition of the biofilms, future work could also investigate changes in community function using metagenomics, metabolomics and metatranscriptomics, in response to different active agents or changes in key environmental parameters.

## Conclusions

This study has successfully developed an oral biofilm model using the CBD seeded with a natural saliva inoculum. The biofilms generated were highly complex and comprised of microbial taxa that are commonly found in dental plaque. In addition, their composition was shown to be reproducible when derived from the pooled saliva of the same panel of individuals. This model will therefore be useful for screening novel antimicrobial agents and also pre- or probiotics that aim to modify plaque composition.

## Additional files

Additional file 1:
**Table showing the alpha diversity of pooled saliva and biofilm samples.** All samples were sub-sampled to 3023 sequences. P - panel; T - time point; S – saliva; SP – PMA-treated saliva; 7d - 7 days incubation; 14d - 14 days incubation; a, b, c - replicate. Additional file 2:
**Dendrogram showing the similarity of biofilm and saliva samples based on community membership (Jaccard index).** P - panel; T - time point; S – saliva; S_P – PMA-treated saliva; 7D - 7 days incubation; 14D - 14 days incubation; a, b, c - replicate. Additional file 3:
**Dendrogram showing the similarity of biofilm and saliva samples based on community structure (thetaYC calculator).** P - panel; T - time point; S – saliva; S_P – PMA-treated saliva; 7D - 7 days incubation; 14D - 14 days incubation; a, b, c - replicate. Additional file 4:
**Bar chart of the predominant genera detected in PMA-treated saliva samples.** The chart shows the mean relative abundances of genera that were detected in all three of the pooled saliva samples from different panels. Genera shown are those with mean relative abundances of > 1%. Error bars show the standard error of the mean (SEM). Additional file 5:
**Table showing the classification of sequences to the species level in each sample.** The table shows the relative abundances of the phylotypes in the different samples. P - panel; T - time point; S – saliva; S_P – PMA-treated saliva; 7D - 7 days incubation; 14D - 14 days incubation; a, b, c - replicate.
