# Supplementary figures and images for: Development and pyrosequencing analysis of an in-vitro oral biofilm model
Source: BMC Microbiol. 2015 Feb 10;15:24. doi: 10.1186/s12866-015-0364-1 (PMC4332733; doi:10.1186/s12866-015-0364-1)

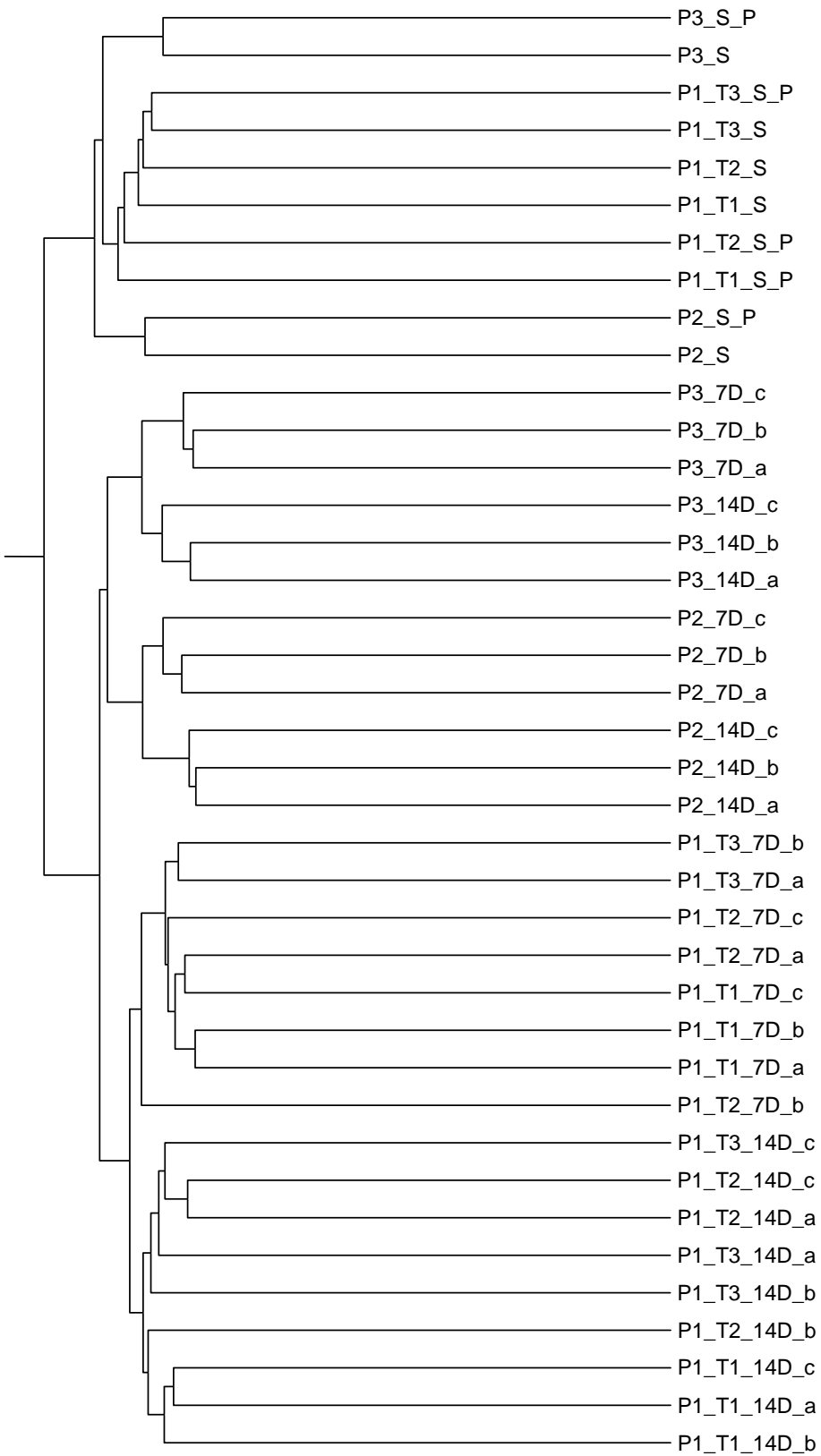

0.1

Supplement: Additional file 2: — Dendrogram showing the similarity of biofilm and saliva samples based on community membership (Jaccard index). P - panel; T - time point; S – saliva; S_P – PMA-treated saliva; 7D - 7 days incubation; 14D - 14 days incubation; a, b, c - replicate. [file 12866_2015_364_MOESM2_ESM.pdf]

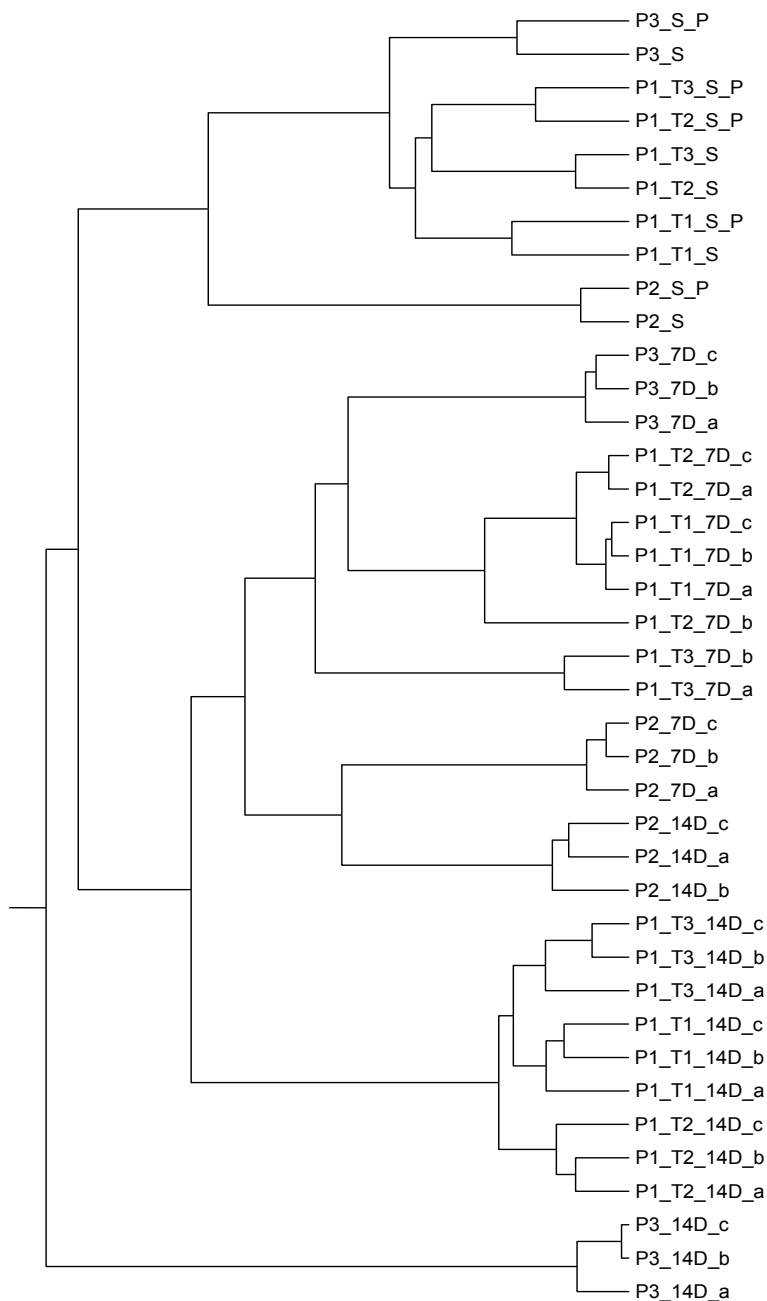

0.1

Supplement: Additional file 3: — Dendrogram showing the similarity of biofilm and saliva samples based on community structure (thetaYC calculator). P - panel; T - time point; S – saliva; S_P – PMA-treated saliva; 7D - 7 days incubation; 14D - 14 days incubation; a, b, c - replicate. [file 12866_2015_364_MOESM3_ESM.pdf]

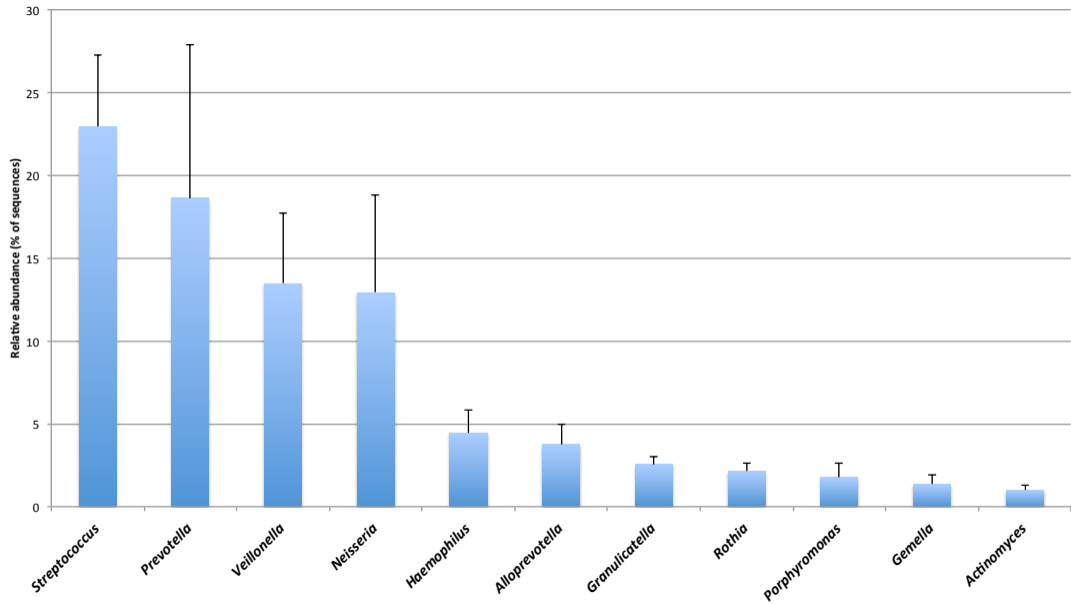

Supplement: Additional file 4: — Bar chart of the predominant genera detected in PMA-treated saliva samples. The chart shows the mean relative abundances of genera that were detected in all three of the pooled saliva samples from different panels. Genera shown are those with mean relative abundances of > 1%. Error bars show the standard error of the mean (SEM). [file 12866_2015_364_MOESM4_ESM.pdf]
